# Supplementary material for: Mercury Exposure and Its Health Effects in Workers in the Artisanal and Small-Scale Gold Mining (ASGM) Sector—A Systematic Review
Source: Int J Environ Res Public Health. 2022 Feb 13;19(4):2081. doi: 10.3390/ijerph19042081 (PMC8871667; doi:10.3390/ijerph19042081)
Supplement: Supplementary file 1 [file ijerph-19-02081-s001.zip › S1_Evaluation_Risk_Of_Bias.pdf]

Supplemental Table S1
Bias assessment

Study:	
Grade of evidence	
**Internal validity – bias**[1]	
Are participants blinded to their intervention?	n.a. (not applicable)[2]
Are staff personnel measuring the results blinded?	
Is there any hint for data dredging?	
Is the length of follow-up equal between the groups?	n.a. (not applicable)[2]
Are the statistical methods adequate?	
Is the compliance with interventions reliable?	n.a. (not applicable)[2]
Are the main outcome measures valid and reliable?	
**Internal validity – confounder**[1]	
Is each group recruited from the same population?	
Is each group in recruited over the same time?	
Are participants randomized to groups **(random sequence generation)**?	n.a. (not applicable)[2]
Is the randomization concealed for participants and staff **(allocation concealment)**?	n.a. (not applicable)[2]
Is there adequate adjustment for confounders?	
Are losses of follow-up mentioned?	n.a. (not applicable)[2]
**Performance bias**[3]	
Is there evidence for a different treatment of the groups? Are participants and personnel blinded (see above)?	
**Detection bias**[3]	
Is there evidence for a **recall bias** of the participants?	
Is there evidence for an **information bias**?	
Is the outcome assessment blinded?	
**Attrition bias**[3]	
Is there evidence for incomplete data?	
**Reporting bias**[3]	
Is there evidence for selective reporting of significant results?	
**Other bias**[3]**:**	

[1] adopted from question 14-26 of:: Downs SH, Black N. The feasibility of creating a checklist for the assessment of the methodological quality both of randomised and non-randomised studies of health care interventions.. Journal of Epidemiology & Community Health 1998;52:377-384.

[2] The question's design for interventional studies does not fit to the given context of expositional studies: the rating n.a. (not applicable) is used and the question was not taken into account for the evaluation

[3] adopted and modified from: Higgins JPT, Altman DG, Sterne JAC (editors). Chapter 8: Assessing risk of bias in included studies. In: Higgins JPT, Green S (editors). *Cochrane Handbook for Systematic Reviews of Interventions* Version 5.1.0 (updated March 2011). The Cochrane Collaboration, 2011. Available from www.handbook.cochrane.org.
