# Supplementary material for: Mercury Exposure and Its Health Effects in Workers in the Artisanal and Small-Scale Gold Mining (ASGM) Sector—A Systematic Review
Source: Int J Environ Res Public Health. 2022 Feb 13;19(4):2081. doi: 10.3390/ijerph19042081 (PMC8871667; doi:10.3390/ijerph19042081)
Supplement: Supplementary file 1 [file ijerph-19-02081-s001.zip › S2_Overall risk of bias.pdf]

Supplemental Table S2
Overall risk of bias

Study	Study design	Overall risk of bias
Afrifa et al. [2017]	Cross-sectional study (XS)	low risk
Afrifa et al. [2018]	Cross-sectional study (XS)	high risk
Rajaee et al. [2015]	Cross-sectional study (XS)	high risk
Mensah et al. [2016]	Cross-sectional study (XS)	high risk
Bose-O'Reilly et al. [2010a]	Cross-sectional study (XS)	low risk
Harada et al. [1999]	Cross-sectional study (XS)	high risk
Bose-O'Reilly et al. [2008]	Cross-sectional study (XS)	low risk
Steckling et al. [2014]	Cross-sectional study (XS)	high risk
Tayrab [2017]	Cross-sectional study (XS)	high risk
Tomicic et al. [2011]	Cross-sectional study (XS)	high risk
Wanyana et al. [2020]	Cross-sectional study (XS)	high risk
Bose-O'Reilly et al. [2010b]	Cross-sectional study (XS)	low risk
Ekawanti and Krisnayanti [2015]	Cross-sectional study (XS)	high risk
Khan et al. [2012]	Cross-sectional study (XS)	high risk
Riaz et al. [2016]	Cross-sectional study (XS)	high risk
Lacerda et al. [2020]	Cross-sectional study (XS)	high risk
Branches et al. [1993]	Case series (IV)	high risk
Harari et al. [2012]	Cross-sectional study (XS)	high risk
Schutzmeier et al. [2016]	Cross-sectional study (XS)	high risk
